# The association between sleep duration and myopia among Chinese school-age students: mediation effect of physical activity

**DOI:** 10.3389/fpubh.2024.1460410

**Published:** 2025-01-03

**Authors:** Haiqing Feng, Yuexia Gao, Na An, Yitong Lu, Jianping Huang, Weiping Yang, Qingyun Lu

**Affiliations:** ^1^Department of Health Management, School of Public Health, Nantong University, Nantong, China; ^2^Nantong Center for Disease Control and Prevention, Nantong, China; ^3^Dafeng People's Hospital, Yancheng, China

**Keywords:** school-age students, sleep duration, myopia, physical activity, mediating effect

## Abstract

**Background:**

This study aimed to investigate the relationship between sleep duration and myopia in school-age students, as well as to observe the role of physical activity as a mediating variable in sleep duration and myopia.

**Methods:**

Using multistage stratified sampling, 26,020 school-age students in Jiangsu Province, ages 7–18, were selected for this cross-sectional survey. Each participant completed a standardized interview in which their were asked about their vision, level of physical activity and average hours of sleep per day over the past month. Visual acuity examinations were conducted by the testing team’s ophthalmology professionals and they were categorized into two groups based on pre-set criteria: myopic and non-myopic. Sleep duration was classified into 3 categories: <8 h/d, 8–10 h/d, >10 h/d. The Pearson’s chi-square tests were used to examine differences in the incidence of myopia among participants. Binary multifactorial logistic regression adjusted for potential confounding variables was used to examine the relationship between myopia and a variety of factors. The mediating effects of physical activity on sleep duration and myopia were analyzed using the AMOS model.

**Results:**

In this study, the incidence of myopia was higher in females than in males, it also increased with age. Those who slept for <8 h/d had the greatest myopia rate (85.69%) compared with those who slept for≥8 h/d (*p* < 0.001). Sleep duration was found to be adversely correlated with myopia (sleep duration = 8–10 h/d: OR = 0.68, *p* < 0.01; sleep duration > 10 h/d: OR = 0.48, *p* < 0.01) after controlling for potential confounders like gender, age, and BMI-z score (Body Mass Index Score). Physical activity at a high intensity not moderate intensity was found to be protective factor against myopia. High-intensity physical activity also acted as a mediator in the negative association between sleep duration and myopia (a, b, c’ all *p* < 0.05).

**Conclusion:**

There is a negative correlation between sleep duration and myopia in school-age students, and that high-intensity physical activity mediates the relationship between sleep duration and myopia.

## 1 Introduction

Myopia is a type of refractive error (1), which is based on the principle that when the eye is in a relaxed state, parallel rays of light are refracted through the refractive system of the eye, and the focus does not fall accurately on the retina, but rather before it, resulting in blurring of the object being viewed (2). By 2050, 84% of Chinese children and adolescents are predicted to be myopic, compared to an average of roughly 50% worldwide (3). Myopia is a significant risk factor for visual health, as evidenced by studies. In mild situations, it can cause retinal shrinkage, and choroidal miniaturization. Serious cases may result in problems such myopic macular degeneration, cataracts, retinal detachment, and even a chance of blindness (4).

Sleep deprivation and lack of physical activity are common in school-age students and both increase the incidence of myopia (5, 6). Myopia is mainly influenced by genetic predisposition, personal habits and environmental factors (7). Education level, outdoor activities, socioeconomic status, and electronic device use are risk factors for myopia (8). Myopia has been linked in several studies to both late and high-quality sleep (9), and lack of sleep duration has long been recognized as a major risk factor for myopia (10). Human and animal correlation studies have demonstrated that a normal circadian cycle maximizes retinal function and removes issues like corneal flatness and refractive error (11, 12). Insufficient sleep affects the ventral striatum’s dopamine D2 receptors, which causes the retinal dopamine pathway to be less activated and the eye axis to elongate, and increased the incidence of myopia (13).

In recent years, physical activity has received increasing attention because of its positive effects on myopia. Physical activity efficiently averted myopia, enhanced students’ visual acuity, and alleviated vision loss, according to a sequential multimediation SEM (Structural Equation Modeling) analysis study conducted among Chinese college students (14, 15). Notably, research has indicated that a sedentary lifestyle raises the risk of myopia and that proper physical activity is associated with a lower incidence of myopia (16). Furthermore, prior research has validated the correlation between physical activity and sleep duration, indicating that more sleep duration is associated with increased physical activity the following day (17). This could be explained by the fact that sleep deprivation increases physical exertion (18), leading to perceived fatigue, which affects next-day exercise capacity and reduces physical activity participation (19). Therefore, based on the discussion between the above variables, we speculate physical activity may be one of the potential mechanisms underlying the association between sleep duration and myopia.

Although it has been recognized that there is an interaction between sleep, physical activity and myopia, however, the majority of earlier research has only highlighted the link between physical activity and myopia (15, 20), without investigating whether myopia is impacted by variations in the level of physical activity. We will go into more detail on the connection between myopia, physical activity, and sleep duration in this study. Furthermore, a mediation effects model will be employed to evaluate the potential mediating role of physical activity and investigate the specific intensity of physical activity that mediates the association between the sleep duration and myopia.

## 2 Materials and methods

### 2.1 Sample selection

This study comes from a part of the Common Diseases and Health Risk Factors Surveillance Programme for Students in Jiangsu Province in 2017. Thirteen cities in the province served as the monitoring region, and multi-stage stratified whole cluster random sampling was employed. Ten of these cities were chosen as provincial-level monitoring sites, while three were chosen as national-level monitoring sites. One metropolitan area and one suburban area were chosen at random from each city for the first stage. The second stage involved the random selection of seven schools from each urban area (two elementary schools, two junior high schools, two senior high schools, and one vocational high school) and five schools from each suburban area (two elementary schools, two junior high schools, and one senior high school). In each city where the national-level monitoring sites existed, one more comprehensive university was added, and monitoring was carried out for them. In the third stage, one to three classes of students were randomly selected from each grade level in the sampled schools. A total of 26,657 people were surveyed in this study. Excluding the ineligible and illogical questionnaires, 26,020 valid questionnaires were collected, with a return rate of 97.61%. The school-age students in the study were aged between 7 and 18 years.

The study was approved by the Ethics Committee of Nantong University, approval number: NO.(2022)04, and informed consent was obtained from the school, the students, and their guardians before the questionnaire was administered.

### 2.2 Methods

#### 2.2.1 Screening of vision

Ophthalmology professionals in the testing team measure the refractive error of the left eye without ciliary paralysis using an automated optometrist. Spherical lens equivalents, which were computed as the spherical lens value plus half of the refractive value, were computed using refractive measurements. The definition of myopia was ≤0.50D. Myopia screening strictly follows the WS/T 663-2020 standard (21).

#### 2.2.2 Sleep duration

The students’ sleep patterns were assessed using a questionnaire, this study only investigated sleep duration and did not assess sleep quality. Questions included “On average, how many hours and minutes of sleep did you get per day in the past month?” After calculating the total sleep time, the sleep time was divided into three main groups based on the sleep time recommended by the American Sleep Foundation for adolescents: <8 h/day, 8–10 h/day and > 10 h/day (22).

#### 2.2.3 Physical activity

We made the decision to use the Chinese version of IPAQ (International Physical Activity Questionnaire) short paper to evaluate the motor status of these school-age students after reviewing pertinent research (23). “On how many days in the past week did you exercise at a moderate or high intensity?,” “How many hours per day on average?,” and “How many minutes?” were the first questions used to gage the intensity of physical activity. In order to assist students in differentiate the level of intensity of their activity during the previous week, examples were also provided. Moderately intense self-perceptions included being able to talk but not singing, and feeling a bit strained but not tired; high intensity self-perceptions included shortness of breath, difficulty speaking, extreme strain, and potentially increased perspiration. Next, the data were cleaned and screened for outliers, then data were truncated and physical activity levels were calculated, and finally physical activity was categorized into a moderate group (high-intensity physical activity combined ≥3d/week; or moderate-intensity physical activity combined large ≥5d/week; or 3 intensities combined on ≥5 days with a total weekly physical activity level of ≥600 MET-min/week; Metabolic Equivalent of Task-min/week); and a high group (all types of high-intensity physical activity totaling ≥3d and a total weekly force activity level of ≥1,500 MET-min/w; or 3 intensities of physical activity totaling ≥7d and a total weekly force activity level of ≥3,000 MET-min/w).

#### 2.2.4 Covariates

We incorporated sex, age and BMI as confounding factors. Body mass index *z*-values were used in the regression analyses instead of body mass index because they eliminate sex-age specificity between obesity (24). The questionnaire also contained a variety of covariant variable, including the hours a day spent on homework, watching television, accessing the internet. We chose to set a 2-h screen time threshold based on pertinent research (25).

### 2.3 Statistical analysis

Statistical software SPSS26.0 and AMOS26.0 were used to analyze the data. Qualitative data were expressed as number of cases and percentages, and quantitative data were expressed as mean ± standard deviation. The Pearson’s chi-square test was employed to identify variations in the occurrence of myopia across participants with varying attributes. After controlling for confounding factors, the association between myopia and different covariates were examined using binary multifactor logistic regression. The prerequisites for the use of multifactor regression analysis are that the dependent variable is categorical, the observations are independent of each other, there is a linear relationship between the independent and dependent variables, there is no multicollinearity, and the sample size is sufficient. Structural equation modeling was used to analyze mediating effects; and Bootstrap method was used to test the significance of mediating effects. Bootstrap sampling method was used for 5,000 samples and 95% confidence intervals were calculated for the significance of effect values and mediating effects *p* < 0.05 was considered as statistically significant difference. The prerequisites for the use of mediation models are the presence of mediating variables, a clear chronological order, supported by theoretical grounds as well as significant regression coefficients.

## 3 Results

### 3.1 Descriptive analysis and correlation analysis between variables were performed

This investigation includes 26,020 participants with data from eyesight exams and sleep duration. The study participants’ fundamental demographic information are compiled in Table 1 based on whether or not they have myopia. Among 26,020 children and adolescents, their myopia rate increased with grade level. Myopia was more common in women (82.92%) than in men (76.92%). Generally speaking, the myopic group was older and had a higher BMI-z score than the non-myopic population. In the region, urban students (81.97%) had a higher prevalence of myopia than rural pupils (76.92%). The individuals who slept less than 8 h per day had the highest myopia rate (85.69%), followed by those who slept between 8 and 10 h per day (71.31%), and those who slept more than 10 h per day (60.83%) had the lowest myopia rate.

**Table 1:** Basic demographic characteristics of study participants by whether or not they were myopic.

Variables	Number of participants (*n* = 26,020)	Non-myopia cases (*n* = 5,239)	Myopia cases (*n* = 20,781)	Myopia rates (%)	*p* value
**Age**					<0.001
<13	12,739	3,854	8,885	69.75	
13–16	9,464	1,033	8,431	89.08	
16–18	3,817	352	3,465	90.78	
**Sex**					<0.001
Male	13,239	3,056	10,183	76.92	
Female	12,781	2,183	10,598	82.92	
**Area**					<0.001
City	15,173	2,735	12,438	81.97	
Village	10,847	2,504	8,343	76.92	
**Grade**					<0.001
Primary	7,081	2,793	4,288	60.56	
Junior high	9,510	1,583	7,927	83.35	
Senior high	9,242	848	8,394	90.82	
University	187	15	172	91.98	
**BMI**	20.93 ± 0.02	20.11 ± 0.05	21.13 ± 0.03		<0.001
**BMI-z score**	−0.00 ± 0.01	−0.21 ± 0.01	0.05 ± 0.01		<0.001
**Sleep duration (h/d)**					<0.001
<8	16,698	2,390	14,308	85.69	
8–10	7,660	2,198	5,462	71.31	
>10	1,662	651	1,011	60.83	
**Moderate-intensity physical activity**					<0.001
Yes	15,377	3,401	11,976	77.88	
No	10,643	1,838	8,805	82.73	
**High-intensity physical activity**					<0.001
Yes	10,034	2,387	7,647	76.21	
No	15,986	2,852	13,134	82.16	
**Daily homework hours (h/d)**					<0.001
<3	21,768	4,715	17,053	78.34	
≥3	4,252	524	3,728	87.68	
**Daily hours of television viewing (h/d)**					<0.001
<2	23,050	4,555	18,495	80.24	
≥2	2,970	684	2,286	76.97	
**Daily hours of Internet access (h/d)**					<0.001
<2	22,170	4,590	17,580	79.30	
≥2	3,850	649	3,201	83.14	

The presence of myopia (normal = 0, myopia = 1) was used as the dependent variable, sex (male = 0, female = 1), age (<13y = 0, 13–16y = 1, 16–18 = 2), BMI z-score as confounding factors, area (city = 0, village = 1), daily homework hours (≥3 h/d = 0, <3 h/d = 1), daily hours of television viewing (≥2 h/d = 0, <2 h/d = 1), daily hours of Internet access (≥2 h/d = 0, <2 h/d = 1), sleep duration (>10 h/d = 0, 8–10 h/d = 1, <8 h/d = 2), moderate-intensity physical activity (yes = 0, no = 1), and high-intensity physical activity (yes = 0, no = 1) served as the independent variables for the multifactor logistic regression analyses. Four models in all were employed. The study’s findings demonstrated that, even after controlling for variables including gender, age, and BMI z-score, the daily homework hours was a risk factor for myopia and positively correlated with the prevalence of the condition. High-intensity physical activity was protective against myopia and had a negative correlation with its incidence. There was no statistically significant difference observed in the moderate-intense physical activity, daily hours of Internet access, or daily hours of television viewing (*p* > 0.05) (Table 2).

**Table 2:** Logistic regression analysis of the relationship between sleep duration and myopia in school-age students.

Independent variable	Myopia							
	Model 1	*p*	Model 2	*p*	Model 3	*p*	Model 4	*p*
**Sleep duration (h/d)**								
>10	**0.48 (0.42–0.53)**	**<0.01**	**0.49 (0.44–0.55)**	**<0.01**	**0.49 (0.44–0.55)**	**<0.01**	**0.50 (0.44–0.56)**	**<0.01**
8–10	**0.68 (0.63–0.73)**	**<0.01**	**0.70 (0.65–0.75)**	**<0.01**	**0.70 (0.65–0.76)**	**<0.01**	**0.70 (0.65–0.76)**	**<0.01**
**<**8	1.00		1.00		1.00		1.00	
**Gender**								
Male	0.65 (0.61–0.69)	<0.01	0.65 (0.61–0.69)	<0.01	0.65 (0.61–0.69)	<0.01	0.65 (0.61–0.70)	<0.01
Female	1.00		1.00		1.00		1.00	
**Age**								
<13	0.31 (0.27–0.35)	<0.01	0.32 (0.29–0.37)	<0.01	0.33 (0.29–0.37)	<0.01	0.33 (0.29–0.38)	<0.01
13–16	0.85 (0.75–0.97)	0.01	0.86 (0.75–0.97)	0.02	0.86 (0.76–0.98)	<0.01	0.86 (0.76–0.98)	<0.01
16–18	1.00		1.00		1.00		1.00	
**BMI z-score**	1.10 (1.07–1.14)	<0.01	1.10 (1.06–1.14)	<0.01	1.10 (1.06–1.14)	<0.01	1.10 (1.06–1.14)	<0.01
**Area**								
City			1.09 (1.02–1.16)	0.01	1.10 (1.03–1.17)	0.01	1.10 (1.03–1.17)	0.01
Village			1.00		1.00		1.00	
**Daily homework hours (h/d)**								
≥3			1.38 (1.24–1.52)	<0.01	1.38 (1.24–1.52)	<0.01	1.37 (1.24–1.52)	<0.01
<3			1.00		1.00		1.00	
**Daily hours of television viewing (h/d)**								
≥2			1.09 (0.91–1.31)	0.33	1.10 (0.92–1.31)	0.32	1.10 (0.92–1.31)	0.32
<2			1.00		1.00		1.00	
**Daily hours of Internet access (h/d)**								
≥2			1.03 (0.93–1.14)	0.58	1.02 (0.93–1.13)	0.67	1.02 (0.93–1.13)	0.66
<2			1.00		1.00		1.00	
**Moderate-intensity physical activity**								
Yes					0.99 (0.91–1.08)	0.83	1.00 (0.91–1.10)	0.95
No					1.00		1.00	
**High-intensity physical activity**								
Yes							**0.88 (0.81–0.96)**	**<0.01**
No							1.00	

Model 1: adjusted for gender, age and BMI z-score. Model 2: adjusted for gender, age, BMI z-score, area, homework time, TV time, Internet time, and sleep time. Model 3: adjusted for gender, age, BMI z-score, area, homework time, TV time, Internet time, sleep time, and moderate-intensity physical activity. Model 4: adjusted for gender, age, BMI z-score, area, homework time, TV time, Internet time, sleep time, moderate-intensity physical activity and high-intensity physical activity. The values in bold in the table indicate factors that are significantly associated with the risk of myopia. These data highlight the fact that sleep duration and high- intensity physical activity are associated with myopia even after controlling for many covariates.

### 3.2 Mediation analyses

**Figure 1:**
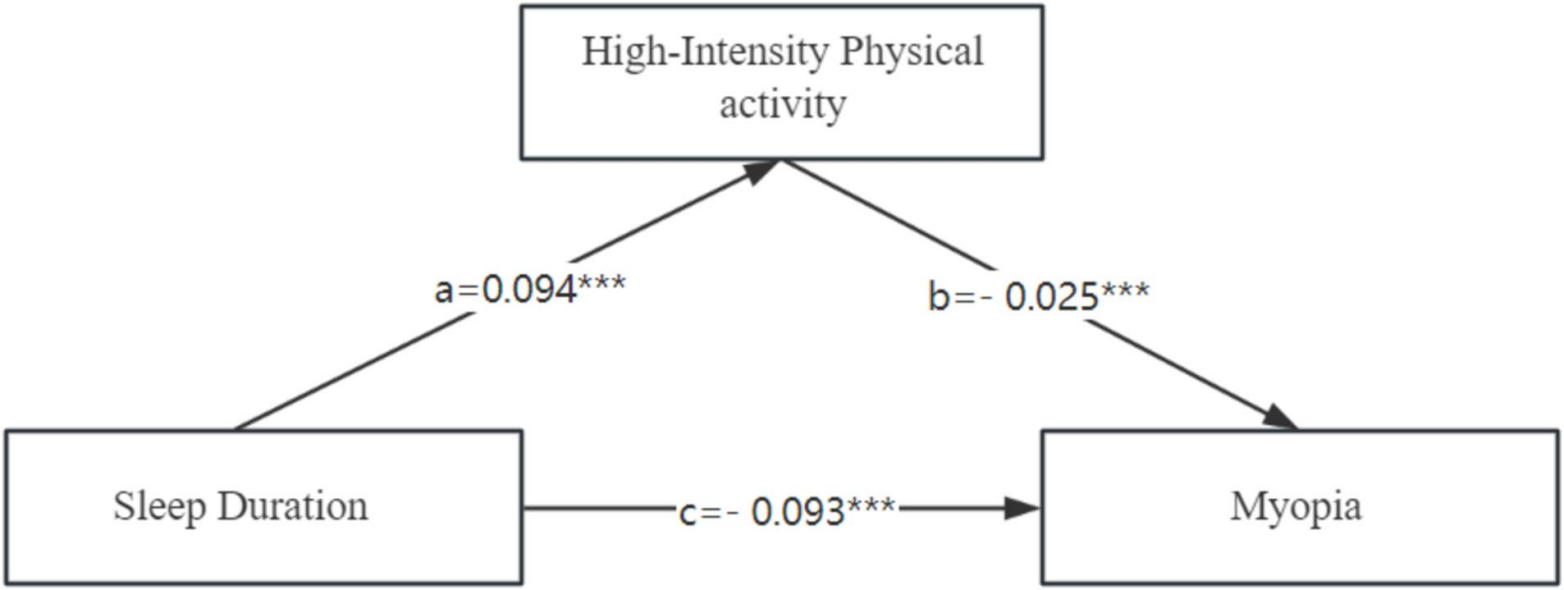
Structural equation modeling diagram. ***p < 0.001.

We explored the relationship between physical activity intensity, sleep duration and myopia by using moderate-intensity physical activity and high-intensity physical activity as mediating variables, respectively. Regression analysis revealed that moderate-intensity physical activity was unrelated to myopia, which means that it cannot be utilized as a mediating variable. The mediation effects analysis results demonstrated that the Bootstrap 95% confidence intervals (−0.160, −0.130) for the total effect caused by myopia and sleep duration did not contain a value of 0, indicating a significant effect of sleep duration on myopia (the effect value was −0.147); the Bootstrap 95% confidence intervals for the direct effect of sleep duration on myopia (−0.156, − 0.127) did not contain a value of 0, indicating a significant direct effect of sleep duration on myopia (effect value of −0.142, accounting for 96.6% of the total effect); the Bootstrap 95% confidence interval for the mediating effect of high-intensity physical activity between sleep duration and myopia (−0.005, −0.002) did not contain a value of 0, suggesting a significant mediating effect. Thus, our hypothesized mediation model is valid, with a direct negative effect of sleep duration on the occurrence of myopia (path coefficient = −0.093), a positive effect of the independent variable sleep duration on the mediator variable high-intensity physical activity (path coefficient = 0.094), and a negative effect of high-intensity physical activity on the occurrence of myopia (path coefficient = −0.025). Sleep duration affects the occurrence of myopia through high intensity physical activity with a mediating effect value of 3.4%. Table 3 displays the effect values for the aforementioned routes, and Figure 1 displays the mediation model.

**Table 3:** Bootstrap test for mediating effects of high-intensity physical activity.

Effect	Effect value	*S.E.*	Percentage of total effect (%)	*p*	Boot95%CI
Total effect (c)	−0.147	0.004	–	<0.001	(−0.160, −0.130)
Direct effect (c’)	−0.142	0.004	96.6	<0.001	(−0.156, −0.127)
Indirect effect	−0.005	0.001	3.4	<0.001	(−0.005, −0.002)

After controlling for the confounders (gender, age and BMI-z score), the model fit index RMR (Root Mean Square Error) = 0.016, which is lower than the acceptable minimum value of 0.05. GFI (Goodness-of-Fit Index) = 0.986, and AGFI (Adjusted Goodness-of-fit Index) = 0.957, which are higher than 0.9, satisfying the criteria for the value of the fit index, it can be seen that the fit of the model to the data is within the acceptable range, indicating that the results of the analysis are acceptable.

## 4 Discussion

This study revealed that sleep duration was negatively associated with the risk of myopia in school-age students, and further analysis of the type of physical activity indicated that only high-intensity physical activity mediated the association between sleep duration and myopia.

Myopia is very common in Jiangsu Province among school-age students. Additionally, senior high school students had a higher detection rate of myopia than primary and junior high school students. The probable reason is that students in Jiangsu Province, which has one of China’s highest levels of educational growth, are under a lot of pressure to perform well academically, which causes them to put in extended study sessions and utilize their eyes excessively. Additionally, students’ study assignments and time typically increase with grade level, resulting in greater close eye time. Myopia was far more common in women than in men, which is in line with the findings of other studies (26). According to Maciej and other academics (27), dietary practices, extended periods of close work, and family genetics account for the majority of the gender gap. There were differences in the prevalence of myopia between school-age children in urban and rural areas. These differences could be attributed to a variety of factors, including education and outdoor time (28). However, it has also been suggested that urban air pollution may hasten the development of myopia (29).

According to this study, watching more than 2 h of television a day was not associated with the development of myopia. Our results are supported by a Norwegian refractive study that involved 224 engineering students over the course of 3 years and found no link between television consumption and the development of myopia (30). The phenomena may be explained by the fact that kids these days have to do more homework and watch less television overall. Another explanation is that myopia is becoming less common as TV screens get larger and people watch them from a greater distance (31). Notably, we also did not discover any correlation between the amount of time spent on the Internet every day and myopia. In a cross-sectional study involving 836 local primary school students and a questionnaire format, researchers in Serbia came to the same conclusion as us: there was no significant correlation between daily Internet use and myopia (32). This may be the result of parents being more conscious of their kids’ visual issues and preventing myopia early on by helping them with their posture online, getting them up and moving at the right times, etc. Bener and other academics, however, contend (33) that excessive Internet use is the primary cause of vision loss, with prolonged usage being more detrimental to the eyes. The erratic relationship may result from the many methods used to measure Internet usage as well as the disparate target audiences.

Our findings are consistent with the results of a cross-sectional study in China on the relationship between sleep duration and myopia in school-aged students (3), that is, increased sleep duration was associated with a reduced risk of myopia in school-aged children, mainly because the major neurotransmitter DA (Dopamine) in the retina releases NO (Nitric Oxide) through the choroid or retina, which thickens the choroidal layer and prevents the growth of the eyeball, slowing down the progression of myopia (34). Myopia results from altered choroidal rhythms caused by circadian rhythm disruption brought on by sleep deprivation (35). Remarkably, a subgroup study revealed (36) that the relationship between sleep duration and myopia was specific to Asian nations, with no association found in European nations. This could be connected to factors like racial disparities, lifestyle choices, and methods of education (37). Firstly, students in Asian nations—particularly China, South Korea, and Japan—often struggle with a great deal of coursework, spending a lot of time studying to get into a reputable university (38). Prolonged near-eye use leads to a decrease in sleep duration and an increase in myopia rates. In contrast, countries such as Europe may pay more attention to study-life balance, they will spend more time on outdoor sports and less time on homework, so the relationship between sleep duration and myopia may be less significant. Secondly, because of increasingly challenging or unfinished homework, students may suffer from psychological issues like anxiety, which will shorten their sleep duration and raise their risk of developing myopia. Additionally, because of the intense academic competition in Asian nations, students will play with electronic devices in the evenings to escape from the real pressure and feel a sense of accomplishment in virtual time. This will also result in fewer hours of sleep and an increase in the rate of myopia. Furthermore, while the exact mechanism between sleep duration and myopia remains unknown, this study highlights that solely the duration of sleep, not the quality of sleep, is linked to myopia (39). This is in line with other findings that have been noted in children and adolescents.

In the present study, we discovered that the degree of high-intensity physical activity increased in concert with the duration of sleep. Sleep and physical activity have been demonstrated to be significantly correlated, with having a restful night’s sleep increases one’s level of physical activity (40). Lack of sleep causes the inflammatory response to increase following physical activity (41), which raises post-exercise fatigue (18). Furthermore, some researchers believe that sleep deprivation can lead to a drop in the body’s core and hand temperatures, which may prompt an individual to stop exercising (19). A study conducted in the Chinese province of Taiwan indicated that engaging in at least 60 min of moderate-to-intense physical activity each day lowered the incidence of myopia in school children (42). When compared to sedentary lives, frequent physical activity lowers the prevalence of myopia, according to another study on 661 white children aged 12 to 13 years (43). Dynamic exercise leads to a drop in intraocular pressure, which helps prevent myopia (44–46). Hypercapnia and hyperventilation during exercise encourage increased atrial fluid outflow, which in turn encourages a drop in venous pressure and a lowering of IOP (Intraocular Pressure) (47). Several studies have suggested that outdoor exercise has a protective effect against myopia. The main mechanism is that outdoor sunlight can increase choroidal blood perfusion, promote choroidal thickening, and regulate scleral remodeling and eye growth (48, 49). In China, since schooling takes up most of the time in students’ daily lives, students’ physical activities tend to be concentrated on campus. Most of their physical activities and exercises take place in outdoor spaces, such as open-air playgrounds. Therefore, it is possible that the interaction between physical activities and outdoor sunlight has an effect on myopia. This study should clarify outdoor and indoor activities in the future to avoid the interference of confounding factors.

Interestingly, in the current study neither sleep nor myopia were shown to be correlated with moderate-intensity physical activity, however, only high-intensity physical activity was found to mediate the association between the two. This outcome was most likely caused by the following: (1) Research by Schuman and other researchers has demonstrated a relationship between the degree of respiratory resistance and the rise of IOP (50). When compared to moderate-intensity physical activity, high-intensity activity requires higher levels of carbon dioxide, which raises respiratory rate. This, in turn, lowers intraocular pressure and lessens myopia (51). When engaging in high-intensity physical activity as opposed to moderate-intensity physical activity, self-regulatory mechanisms are more effective (52, 53). (2) Lactate and IOP have a negative association (54), lactate may be a significant factor in the intake and outflow of aqueous humor, and intense activity causes lactate levels to rise more quickly than moderate-intensity activity does. (3) By boosting slow-wave sleep and decreasing sleep latency, physical activity can enhance sleep. Only high-intensity physical activity is linked to homeostatic restorative processes during the first sleep cycle (55) and has the capacity to lengthen slow wave sleep (56). Therefore, moderate-intensity physical activity may serve as a springboard for high-intensity activity for school-age students, and it is possible to encourage longer and higher-quality sleep. (4) Children’s myopia may be lessened by high-intensity activity because it may increase the volume of blood in circulation, which will enhance the blood flow to the muscles involved in the eyes. It’s possible that moderate-intensity exercise will not stimulate the eyes enough to noticeably increase blood flow.

There are several restrictions on our investigation. First, since ciliary muscle paralyzing drugs were not utilized during the examination of visual acuity, the prevalence of myopia may have been overstated. A comparison between dilated and non-cycloplegic refraction measurements could be conducted in future studies to further validate and understand the differences between the two methods. Second, we are unable to determine the exact causal association between myopia, sleep, and physical activity components due to the cross-sectional survey approach utilized in this study. This survey also had recollection bias and was self-reported. As a result, going forward, more objective metrics and significant variables ought to be applied. Future research should include more components and use more specialized equipment to study the association between variables, as myopia is influenced by a multitude of factors, including lifestyle choices, time spent outdoors in the sun, and hereditary factors.

The economic and cultural standards of Jiangsu Province are comparatively high in China, and the academic demands placed on age-school children in Jiangsu are higher than those of kids in other provinces. As a result, it may be difficult to extrapolate our findings to other populations. Nevertheless, the study’s information bias was mitigated by the fact that a ophthalmology professionals determined the participant’s myopia. The results of this study have some reference value for school-age students in similar provinces or regions because it is a survey of a sizable sample population throughout the entire province and because sampling took into consideration the impact of social and economic disparities among regions on health resources.

## 5 Conclusion

This study demonstrates a negative relationship between myopia and sleep duration, which is mediated by high-intensity physical activity. Therefore, Health policymakers should enforce more stringent measures to prevent and control myopia, establish a system for monitoring the vision and sleep of children and adolescents, and perform routine vision examinations and sleep evaluations in order to identify issues early and take appropriate action. Families must encourage their kids to have healthy eye habits and increase the duration of time they spend exercising, particularly at greater intensities. On the other hand, schools must improve their teaching of eye health and lessen the academic load on children.

## Advantages

The information bias of this study was mitigated by the fact that participants’ myopia was determined by the testing team’s ophthalmology professionals. The results of this study are useful for school-age students in similar provinces or regions.

## Limitations

Firstly, this study used a cross-sectional study, which collected data at a single point in time, making it impossible to determine the temporal order between variables and difficult to determine causal relationships. And participants may have relied on memory to report past behaviors or experiences, which could lead to recall bias and affect the accuracy of the data. As data may be collected differently in different locations, the quality and accuracy of the data may also be affected to varying degrees. Secondly, no ciliary muscle paralyzing medication was used to check visual acuity, which may have exaggerated the prevalence of myopia.

## Data Availability

The datasets presented in this article are not readily available because the datasets used to support the findings of this study are available from the corresponding author on reasonable request. These data are not publicly available due to privacy or ethical constraints. Requests to access the datasets should be directed to fenghaiqing1105@163.com.
